# The deep learning-based physical education course recommendation system under the internet of things

**DOI:** 10.1016/j.heliyon.2024.e38907

**Published:** 2024-10-03

**Authors:** Aiyuan Zhen, Xin Wang

**Affiliations:** a School of Physical Education, Shanghai Normal University, Shanghai, 200234, China; b Faculty of Physical Education, China West Normal University, Nanchong, 637000, China

**Keywords:** course recommendation, Internet of Things, Deep learning, Generative adversarial networks, Conditional feature generative adversarial networks

## Abstract

This study aims to propose a deep learning (DL)-based physical education course recommendation system by combining the Internet of Things (IoT) technology and DL, to improve the accuracy and personalization of recommendation. Firstly, IoT devices such as smart bracelets and smart clothing are used to monitor students' physiological data in real-time, and IoT sensors are utilized to sense the environment around students. Secondly, IoT devices capture students' social interactions with their peers, recommending socially oriented courses. Meanwhile, by integrating IoT data with students' academic data, course recommendations are optimized to match students' learning progress and schedule. Finally, Generative Adversarial Network (GAN) models, especially the improved Regularization Penalty Conditional Feature Generative Adversarial Network (RP-CFGAN) model, deal with data sparsity and cold start problems. The experimental results show that this model performs well in TopN evaluation and is markedly enhanced compared with traditional models. This study denotes that integrating IoT technology and GAN models can more accurately understand student needs and provide personalized recommendations. Although the model performs well, there is still room for improvement, such as exploring more regularization techniques, protecting user privacy, and extending the system to diverse platforms and scenarios.

## 1 Introduction

As an effective tool for information filtering and personalized services, recommendation systems have become an important way to solve the information overload problem [[1], [2], [3]]. Recommendation systems are widely used in fields, such as e-commerce, social media, movies, and music [4]. However, in physical education (PE), especially in the recommendation of PE courses, the application of recommendation systems is relatively limited, and there is huge potential and demand.

The design and recommendation of traditional PE courses usually adopt a universal model, which cannot fully meet each student's personalized needs and preferences. With the development of Internet of Things (IoT) technology, more and more sports and fitness devices (such as smart bracelets and insoles) have generated a large amount of exercise-related data, including step count, heart rate, and calories burned [[5], [6], [7]]. These data provide valuable information resources for implementing personalized PE course recommendations. However, traditional recommendation methods cannot fully explore the potential patterns and associations behind these data due to the complexity and personalization of data. Therefore, useful information can be better extracted from massive motion data to achieve more accurate personalized recommendations using Deep Learning (DL) technology, especially models, such as Generative Adversarial Networks (GANs) [8,9]. Personalized course recommendations based on personal behavior data can be achieved by combining IoT with recommendation systems to meet students' learning needs and sports interests.

In this context, this study explores how to combine IoT and DL to construct an intelligent personalized sports course recommendation system to meet students' personalized needs in PE course selection. It is expected to provide more accurate and targeted PE course recommendations for each student to improve PE's quality and learning effectiveness by analyzing student behavior data deeply, mining hidden information behind the data, and combining advanced DL.

The core goal of this study is to investigate how to use IoT data and DL to build an effective personalized sports course recommendation system. To ensure the depth and practicality of the research, the focus is on the following aspects. The DL generation model is used to optimize the extended model Conditional Feature Generative Adversarial Network (CFGAN) of the GAN model and create a collaborative filtering generative adversarial model (CFGAN with “1-Reconstruction” Regularization Penalty (RP-CFGAN)) embedded with dual “1-Reconstruction” RP terms to more accurately predict students' course preferences and adaptability. IoT is integrated into recommendation systems to collect and organize student behavior data from IoT devices to provide a foundation for personalized modeling. The research innovation lies in optimizing CFGAN, introducing a dual “1-Reconstruction” RP, and creating a CFGAN model based on it. This is done to make the recommendation system more creative and personalized, thus improving the accuracy and customer satisfaction of the course recommendation system.

Through the above objectives, this study can furnish students with more targeted and attractive online course selection suggestions, thereby improving PE's quality and learning effectiveness.

The novelty of this study is primarily manifested in several aspects. This study is the first to combine IoT technology with DL for personalized recommendation of PE courses. By leveraging IoT devices to monitor students' physiological data and environmental information in real-time, combined with DL technology, especially GANs, students' needs and preferences are analyzed and understood, thus providing more accurate course recommendations. Subsequently, the dual “1-Reconstruction” RP term is innovatively introduced based on the traditional CFGAN, forming the RP-CFGAN model. This improvement not only enhances the accuracy of the recommendation system but also addresses issues of data sparsity and cold start, offering new insights for the research and application of recommendation systems. Additionally, the study considers students' physiological data and integrates their social interactions and academic data. Through the fusion of multidimensional data, the course recommendation algorithm is optimized. This comprehensive data utilization approach ensures that the recommended results better match students' individual circumstances and learning progress. Finally, through a series of experiments, including hyperparameter influence experiments and comparison experiments with traditional models, the effectiveness of the RP-CFGAN model is verified. The experimental results demonstrate that the RP-CFGAN model markedly outperforms traditional models in recommendation performance, with a notable improvement in accuracy, further confirming the superiority and practicality of the research approach.

The main contributions of this study are as follows. Firstly, the combination of IoT technology and DL is applied to the personalized recommendation system of PE courses. Through real-time monitoring of students' physiological data and environmental information, combined with the GAN model, in-depth analysis and understanding of students' needs and preferences can be provided to offer more accurate course recommendations. Secondly, based on traditional CFGAN, a double “1-Reconstruction” RP term is introduced to form the RP-CFGAN model. This improvement not only improves the accuracy of the recommendation system but also effectively solves the problems of data sparsity and cold start, which provides a new idea for the recommendation system's research and application. In addition, the model design considers students' physiological data, social interaction, and academic data comprehensively. The course recommendation algorithm is optimized through the fusion of multidimensional data, making the recommendation results more in line with the student's situation and learning progress. Overall, this study innovatively improves the PE course recommendation system by integrating IoT technology with DL, providing a more accurate and practical solution for personalized recommendations. These contributions not only enrich the research in the field of recommendation systems but also lay a solid foundation for the future application and development of related technologies.

## 2 Literature review

IoT's interactive and personalized characteristics have brought new ideas for optimizing recommendation systems. A study proposed an IoT-based efficient community recommendation system. This system could diagnose heart diseases and their types and advise patients on physical exercise and dietary plans [10]. Some researchers designed an intelligent background music system based on DL and IoT and applied this system to smart homes. In light of this, a feature extraction algorithm based on mid-level feature structure was proposed to extract the basic features of scene images [11]. Scholars also introduced a hybrid technology that combined implicit collaborative filtering (CF) and ontology to efficiently implement IoT service recommendations, recommending personalized IoT services for users [12]. Recommendation systems could more accurately understand users' interests and needs by analyzing the interaction between users and IoT devices, providing them with more targeted recommendations.

DL methods have achieved remarkable success in the image processing domain. In recent years, researchers have begun to recognize its immense potential in the field of recommendation systems to enhance the efficiency of recommendation algorithms. In this emerging field, DL has demonstrated promising applications. For example, researchers led innovation in combining multi-criteria recommendation and CF with DL and proposed a DL-based multi-criteria CF model. Their research indicated that this model outperformed other state-of-the-art methods on real-world datasets [13]. Similarly, scholars developed a neural network-based music recommendation algorithm that offered high-quality music recommendations to users by automatically analyzing music similarities and achieved significant success, especially across six different types of songs [14]. Another study introduced an innovative interest-based recommendation approach that combined DL and convolutional neural networks. This approach concentrated on the impact of the most similar friendship patterns on recommendations, and considered the diverse influences of user friendships more accurately compared to traditional methods, further improving recommendation accuracy [15]. These cutting-edge research findings showcased the wide-ranging application prospects of DL in the recommendation system field, particularly in innovative applications within CF models. These studies provided crucial theoretical support and inspiration for the research work, encouraging the integration of DL with IoT data to achieve superior personalized course recommendation systems.

As an effective tool for information filtering and personalized services, recommendation systems have become a vital approach to addressing information overload. They are widely used in various fields such as e-commerce, social media, movies, and music. Nevertheless, in the PE field, especially in the recommendation of PE courses, the application of recommendation systems is relatively limited, yet there is significant potential and demand. Traditional PE course design and recommendation typically rely on generic models, which cannot fully meet the personalized needs and preferences of each student. With the development of IoT technology, an increasing number of sports and fitness devices (such as smart bracelets and insoles) generate a large amount of sports-related data, including steps, heart rate, and calories burned. These data provide valuable information resources for implementing personalized PE course recommendations. However, due to the complexity and personalization of the data, traditional recommendation methods fail to fully exploit the underlying patterns and correlations in these data. Hence, using DL technology, especially models like GANs, can extract useful information from massive sports data and achieve more accurate personalized recommendations.

Additionally, attention has been paid to the latest advancements in the domain of recommendation systems. Compared to other studies, this study further explores how combining artificial intelligence (AI) with recommendation system technology can greatly improve the accuracy of the system's recommendations. For example, scholars applied AI to recommendation systems and explored the basic methods and mainstream technologies of recommendation systems [16]. They also investigated how AI could effectively enhance the technological development and application of recommendation systems, reviewing improvements to recommendation systems through AI methods such as fuzzy technology, genetic algorithm (GA), transfer learning, evolutionary algorithms, neural networks, DL, and active learning. Scholars discussed the importance of recommendation systems in addressing information overload, especially in fields like e-commerce, entertainment, and social media, offering guidance for researchers and practitioners to understand new trends and challenges in this area [17]. Additionally, researchers comprehensively examined the latest research on recommendation systems based on Graph Neural Networks (GNNs) and provided a classification method based on the types of information used and the recommendation tasks to classify GNN-based recommendation models [18]. They also systematically analyzed the challenges faced when applying GNN to different types of data and discussed how existing research in this field addresses these challenges.

Based on the above literature review, the application of IoT and DL methods in recommendation systems showed great potential. However, there was insufficient research on PE, especially in the recommendation of personalized PE courses. This study fully utilized IoT and DL to bring breakthroughs in PE, improving the recommendation effect and learning experience of PE courses.

## 3 Research methodology

### 3.1 IOT-BASED course recommendations

**Fig. 1:**
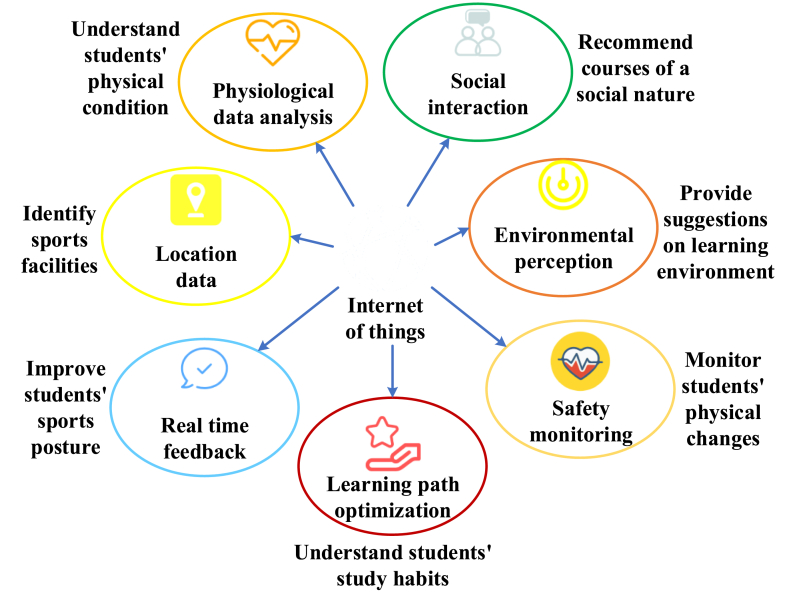
The application of IoT in course recommendations.

This study aims to construct a DL-based PE course recommendation system using IoT technology to better understand students' exercise habits, physical conditions, and preferences by analyzing their physical activity data. Through IoT, diverse data on students' physical activities can be obtained, thereby better understanding their exercise habits, physical condition, and preferences [[19], [20], [21]]. Fig. 1 displays a specific representation.

Fig. 1 illustrates the application of IoT in course recommendations as follows. First, physiological data analysis: IoT devices such as smart wristbands and smart clothing can monitor students' physiological data in real-time, including exercise intensity, body temperature, and heart rate. These data can be combined with course data to help the system better understand the student's physical condition. For example, when the system detects a high heart rate during a PE course, it may indicate the student's interest or full engagement in the course, prompting the system to recommend more related courses. Second, environmental awareness and location data: IoT sensors can perceive environmental information around students, such as temperature, humidity, and light levels. This information can be employed to offer recommendations for a suitable learning environment. For instance, it can recommend outdoor sports classes when the temperature is ideal. By using Global Positioning System (GPS) or other positioning technologies, the system can determine the student's location, helping identify nearby sports facilities and related courses, thus offering personalized course recommendations. Third, social interaction: IoT devices can capture students' social interactions with peers, such as the frequency of exercising together and their social circles. This data can be leveraged to suggest courses or activities with a social focus, thereby encouraging students to participate in sports and recreational activities alongside their peers. Fourth, real-time feedback: IoT devices can furnish instantaneous feedback to aid students in refining their exercise techniques or enhancing their performance. This functionality is facilitated through intelligent exercise equipment, with the system offering tailored recommendations based on the student's real-time data. Fourth, safety monitoring: IoT devices can play a pivotal role in ensuring student safety during physical exertion. For instance, if the device detects a significantly elevated heart rate, the system can alert the student and recommend rest or seeking medical assistance. Lastly, learning path optimization: IoT-generated data can be amalgamated with students' academic records to provide insights into their learning patterns and timetables, enabling the system to adapt and optimize their educational journey accordingly. This assists in optimizing course recommendations to align with the student's learning progress and timetable [[22], [23], [24], [25]].

In summary, IoT can offer students a smarter and more personalized PE course recommendation system, enhancing both their learning and sports experiences. However, when implementing IoT technology, it's essential to consider critical issues such as privacy protection and data security.

### 3.2 A course recommendation model based on GAN

GAN is a DL model composed of two competing neural networks: a generator (G) and a discriminator (D). The fundamental concept of GAN is to train these two networks adversarially, enabling the generator to generate realistic data while the discriminator can accurately distinguish between real and generated data [26,27]. The generator (G) is tasked with transforming a random noise vector into an output resembling real data, progressively refining its parameters to generate increasingly realistic samples. During the training process, the generator's objective is to deceive the discriminator by producing data that closely resembles real data, thus challenging the discriminator's ability to discern between the two [28,29]. Conversely, the discriminator (D) functions as a binary classifier, determining whether input data is real or generated. It continuously adjusts its parameters during training to enhance its ability to distinguish between real and generated data accurately, with the ultimate goal of achieving optimal discrimination between the two data types [[30], [31], [32]].

This study employs GAN to augment the performance of course recommendation systems, enhancing their adaptability to individualized preferences. The reasons for choosing GAN are as follows. First, GAN's capacity for generating diverse data types, such as text and images, lends itself well to generating personalized and varied course recommendations. GAN's generators can create course recommendations, while the discriminator can assess the quality of these recommendations, thus refining the recommendation system. Second, GAN can mitigate sparsity and cold-start issues commonly encountered in course recommendations by filling in data gaps and generating recommendations aligned with user interests, thereby enhancing recommendation accuracy. Third, GAN facilitates enhanced personalization by generating recommendations tailored to individual preferences, surpassing the limitations of traditional CF or content-based methods. This approach ensures that recommendations better align with user interests and requirements. Lastly, GAN enriches recommendation system diversity by generating a broad spectrum of recommendations, mitigating the “filter bubble” phenomenon where users are exposed to repetitive content suggestions. In summary, using GAN can make recommendation systems more creative and personalized, contributing to improved recommendation accuracy and user satisfaction.

**Fig. 2:**
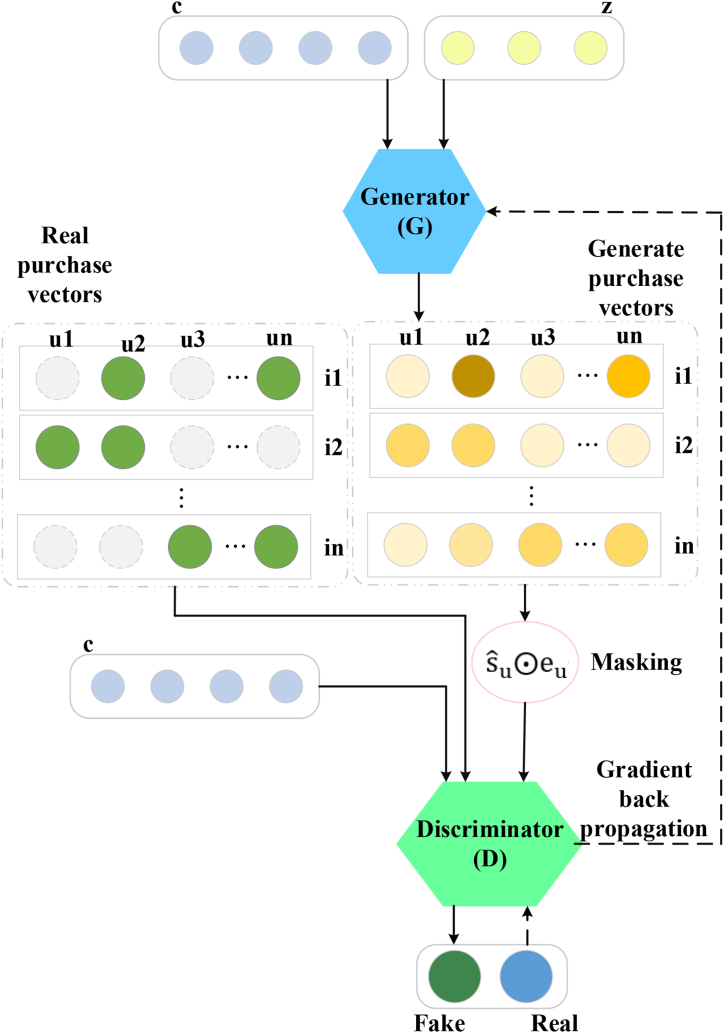
Diagram of CFGAN structure.

The application of GAN in recommendation systems is increasing, with typical examples being Information Retrieval Generative Adversarial Net (IRGAN), Graph GAN, and CFGAN [[33], [34], [35]]. CFGAN performs the best. CFGAN achieves a perfect combination of GAN and CF, allowing the model to maximize the advantages of GAN's game theory during training [36,37]. Its structure is depicted in Fig. 2.

Fig. 2 illustrates the task of the Generator, which is to take a random noise vector as input and transform it into an output resembling real data. The Generator adjusts its network parameters gradually to generate increasingly realistic data samples. During training, the goal of the Generator is to deceive the Discriminator as much as possible, making it difficult to distinguish between the generated data and real data. The Discriminator acts as a binary classifier, tasked with determining whether the input data is real or generated. Throughout training, the Discriminator continuously adjusts its network parameters to enhance its ability to differentiate between real and generated data. The objective of the Discriminator is to accurately distinguish between the two types of data. The training process of GAN can be described as a zero and game. In each training iteration, the Generator and Discriminator compete against each other, updating the network parameters by maximizing the adversarial loss of both the Generator and Discriminator. Specifically, the Generator receives random noise as input and generates data samples. The goal of the Generator is to make the generated samples as close to real data as possible to deceive the Discriminator. The loss function of the Generator is typically measured by the misclassification of the Discriminator, meaning the Generator aims to generate data that the Discriminator mistakenly identifies as real. The Discriminator receives both real and data generated by the Generator and attempts to correctly classify them as real or generated data. The objective of the Discriminator is to accurately differentiate between these two types of data. The loss function of the Discriminator is usually measured by the classification error of the Discriminator on real and generated data. By alternating the training of the Generator and Discriminator, the GAN model continuously optimizes the performance of both, ultimately achieving the goal of generating realistic data samples.

It is assumed that *U* has *m* users, and *I* has *n* items. The interaction matrix between users and items is the real number matrix *S* of *m*∗*n*. A value of one indicates interaction (implicit feedback), while a value of zero indicates no interaction. The true purchase vector of each user *u* is represented as $s_{u}$. $s_{ui}$ indicates whether user *u* has purchased the item *i*, which is one if purchased. Otherwise, it is zero. The set of items purchased by user *u* is represented as $I_{u}$. In the model, the input generator *G* is random noise *z* and user vector *c*. The model's output aims to generate a vector ${\hat{s}}_{u}$. This vector is sparse. ${\hat{s}}_{u}$ and indicator vector $e_{u}$ (dimension *n*) are multiplied by ⊙ element and used as input for discriminator *D*. $e_{ui}$ indicates whether user *u* has purchased item *i*, which is one if purchased. Otherwise, it is zero. The input of discriminator *D* includes the user vector *c* and the processed generated real value vector or the user's real purchase vector. This design enables the model to capture the user's purchasing behavior and generate vectors to ascertain whether the user has purchased a specific item [[38], [39], [40]].

CFGAN includes CFGAN with Zero-Reconstruction (CFGAN_ ZR), CFGAN with Partial-Masking, and CFGAN with Zero-Pair (CFGAN_ZP) [41]. CFGAN's cross-domain recommendation capabilities, robust representation learning, and adaptability to diverse scenarios render it highly effective in addressing recommendation challenges. However, in CFGAN, the values of the output nodes corresponding to all positive samples cannot maintain the same trend of approaching one as the zero RP approaches zero. Additionally, achieving a score close to one for the final recommended item to the user proves challenging in CFGAN [[42], [43], [44]].

To address this issue, CFGAN is improved by embedding a dual “1-Reconstruction” RP term into the CFGAN to form RP-CFGAN. The expression for ‘1-Reconstruction’ is $J^{O}$ can be written as Eq. (1) [45,46]:

(1) $$J^{O}=\gamma *\sum_{i\in O_{u}}{\left(y_{ui}-{\hat{y}}_{ui}\right)}^{2}$$

**Fig. 3:**
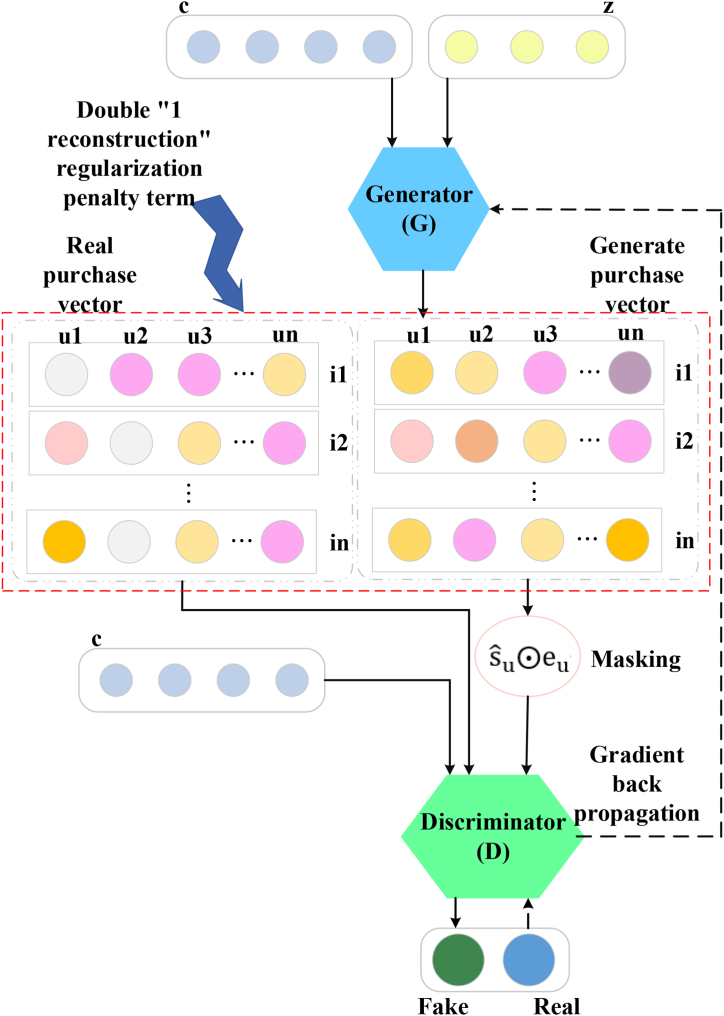
PR-CFGAN framework structure.

γ refers to the “1-Reconstruction” RP coefficient; $O_{u}$ stands for the set of positive samples and a small number of negative samples corresponding to user *u*; ${\hat{y}}_{ui}$ means the true rating of user *u* on item *i*; ${\hat{y}}_{ui}$ represents the prediction score. The framework of RP-CFGAN is displayed in Fig. 3.

RP-CFGAN preserves the ZR and ZP methods in CFGAN and generates RP-CFGAN_ZR and RP-CFGAN_ZP. The physical meaning of RP-CFGAN_ZR is to punish the “all-1 strategy.” The loss functions of G and D are as follows.

(2) $$J^{G}=\sum_{u}[\log\left(1-D\left(\left({\hat{s}}_{u}⨀e_{u}\right)|c_{u}\right)\right)+\delta *\sum_{j}{\left(y_{uj}-{\hat{y}}_{ui}\right)}^{2}+\gamma *\sum_{i}{\left(y_{ui}-{\hat{y}}_{ui}\right)}^{2}]$$

(3) $$J^{D}=-\sum_{u}[\log\;D\left({\hat{s}}_{u}|c_{u}\right)+\log\left(1-D\left(\left({\hat{s}}_{u}⨀e_{u}\right)|c_{u}\right)\right)]$$

In Eq. (2)-Eq. (3), D() represents the probability of determining the truth of a real value vector; D(a|b) indicates the judgment of *a*n under the condition of *b*; $\delta *\sum_{j}\left(*\right)$ means the zero reconstruction RP term; δ refers to the zero RP coefficient, $j\in N_{u}^{ZR\left(t\right)}$. The physical meaning of RP-CFGAN_ZP is to add some interference to the model based on punishing the “all-1 strategy,” thereby strengthing the model. Its loss function and RP-CFGAN_ZR remain consistent.

## 4 Experimental design and performance evaluation

### 4.1 Experimental materials

Currently, most people utilize online learning to acquire knowledge. The Massive Open Online Course (MOOC) platform brings learners from various countries and age groups, furnishing educational researchers with massive user behavior data. The open data warehouse MOOCCube published by Tsinghua University for large-scale online education is used as the original dataset. MOOCCube is an open-source, large-scale data repository designed to serve research related to MOOC. Its distinguishing attributes lie in its extensive scale and diverse data offerings, surpassing those of existing educational resource databases. It includes comprehensive student behavior records, such as learning duration, frequency of study sessions, and intervals of video consumption. MOOCCube encompasses the learning records of nearly 200,000 students, totaling almost 5 million instances of video-viewing and learning activities. This wealth of data serves as a foundational resource for activities ranging from user behavior analysis to modeling and pertinent recommendations. Furthermore, MOOCCube comprises data related to 706 courses and nearly 40,000 videos, meticulously processed to ensure relevance and accuracy. At its core, this repository revolves around knowledge concepts, forging connections between student behavior and course content through the lens of these concepts. This integration culminates in the formation of a cohesive entity known as MOOCCube. One distinctive feature of this dataset is its inclusion of learning behavior data from learners of varying ages and geographical regions participating in MOOC platforms worldwide. The data holds significant reference value for research in online education and the enhancement of personalized course recommendation systems. By conducting analyses and experiments with this dataset, a deeper understanding of learner behaviors and needs can be obtained, ultimately leading to improvements in the performance of personalized course recommendation systems. A dataset containing student behavior records is selected to obtain 14,680 pieces of data that meet the requirements, including 1294 students, 632 courses, and 5916 sessions generated. The data is divided into training, validation, and testing sets in a ratio of 6:2:2.

Precision (P@N), recall (R@N), Normalized Discounted Cumulative Gain (NDCG@N), and Average Reciprocal Rank (RR@N) are used as the TopN evaluation indicators for the recommendation model [[47], [48], [49]]. TopN represents selecting the top N items that are most likely to be favored by the user from all available items or content when a user is given [[[50], [51], [52]]. In this experiment, *N* is taken as 5 and 20 to validate the model. Assuming there are *U* users, *u* represents one of them. The recommended TopN item list for user *u* is *Q* (*u*), and the list of items that user *u* is truly interested in is *W* (*u*). Then:

(4) $$P=\frac{\sum_{u\in U}|Q\left(u\right)\cap W\left(u\right)|}{\sum_{u\in U}|Q\left(u\right)|}$$

(5) $$R=\frac{\sum_{u\in U}|Q\left(u\right)\cap W\left(u\right)|}{\sum_{u\in U}|W\left(u\right)|}$$

(6) $${NDGG}_{N}=\frac{\sum_{i=1}^{N}\frac{2^{{rel}_{i}}-1}{\log_{2}\left(i+1\right)}}{\sum_{i=1}^{\left|REL\right|}\frac{2^{{rel}_{i}}-1}{\log_{2}\left(i+1\right)}}$$

In Eq. (4)-Eq. (6), *N* indicates the size of the recommendation list; ${rel}_{i}$ represents the correlation between the recommendation results of the *i*-th user. |REL | means the set composed of the first *N* results sorted in descending order of relevance [53].

(7) $$RR=\frac{1}{N}\sum_{i=1}^{\left|N\right|}\frac{1}{{rank}_{i}}$$

*N* refers to the number of users; ${rank}_{i}$ represents the ranking of the *i*-th user's first correctly recommended item in the TopN recommendation list [54].

### 4.2 Experimental environment and parameters setting

The experimental environment and parameter settings for the model validation experiment are exhibited in Table 1.

**Table 1:** Experimental environment and experimental parameter settings of model validation.

Parameter	Specification/Size
Operating system	Windows 10
Central processing unit	i5-8400
Graphic processing unit	NVIDIA GTX 1060Ti
Memory	16 GB
DL framework	TensorFlow
Programming language	python3.6
Number of hidden layer nodes for G and D	(50,100,150,200,300)
Learning rate	(0.01,0.005,0.001,0.0005,0.0001)
Optimization method	Adam
Number of hidden layers for G and D	(1,2,3,4,5)
G and D training step ratio $v_{GD}=\frac{G_{step}}{D_{step}}$	(1,2,3,4,5,6,7)
Minibatch	(32,64,128,256)
Activation function	sigmoid

### 4.3 Performance evaluation

#### 4.3.1 Validation of dual ″1-Reconstruction″ effectiveness

**Fig. 4:**
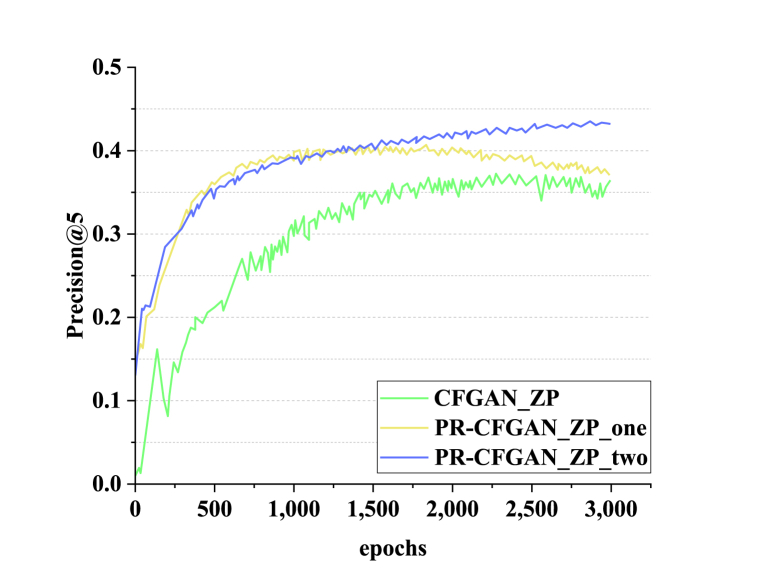
Accuracy learning curve of three methods.

Experiments are conducted on CFGAN_ZP, PR-CFGAN_ZP_two using dual “1-Reconstruction”, and PR-CFGAN_ZP_one using only the positive sample part of “1-Reconstruction” on the same dataset to verify the effectiveness of dual “1-Reconstruction”. Its accuracy P@5 learning curve is plotted in Fig. 4.

Fig. 4 denotes that in terms of curve performance, PR-CFGAN_ZP_two > PR-CFGAN_ZP_one > CFGAN_ZP. It means that introducing the “1-Reconstruction” penalty term at the positive sample position is effective. Meanwhile, after embedding the “1-Reconstruction” penalty term in the positive sample position, adding the “1-Reconstruction” RP term in some negative sample positions can further enhance the model's performance. This is because the introduction of the “1-Reconstruction” penalty term can help the model better distinguish between positive and negative samples. In PR-CFGAN_ZP_two, by using the “1-Reconstruction” penalty term in both the positive sample position and part of the negative sample position, the features of positive samples can be reconstructed more accurately in the model's learning process, thus improving the model's performance. In contrast, PR-CFGAN_ZP_one also uses “1-Reconstruction”, but it is limited to the positive sample portion, resulting in slightly lower performance than PR-CFGAN_ZP_two. Moreover, CFGAN_ZP does not introduce a “1-Reconstruction” penalty term, so it performs the worst in terms of performance. In conclusion, these experimental results show that in the DL-based PE course recommendation system for IoT, the adoption of a double “1-Reconstruction” strategy can effectively enhance the model's recommendation accuracy and performance, thus providing important empirical support for optimizing the course recommendation system.

#### 4.3.2 The impact of key hyperparameters

Based on RP-CFGAN_ZR and RP-CFGAN_ZP methods, the impact of the ratio $v_{GD}$ of training steps for key hyperparameters G and D, sampling ratio $S^{ZR}$ and $S^{PM}$, “1-Reconstruction” RP coefficient γ, and zero RP coefficient δ on the model is studied.

**Fig. 5:**
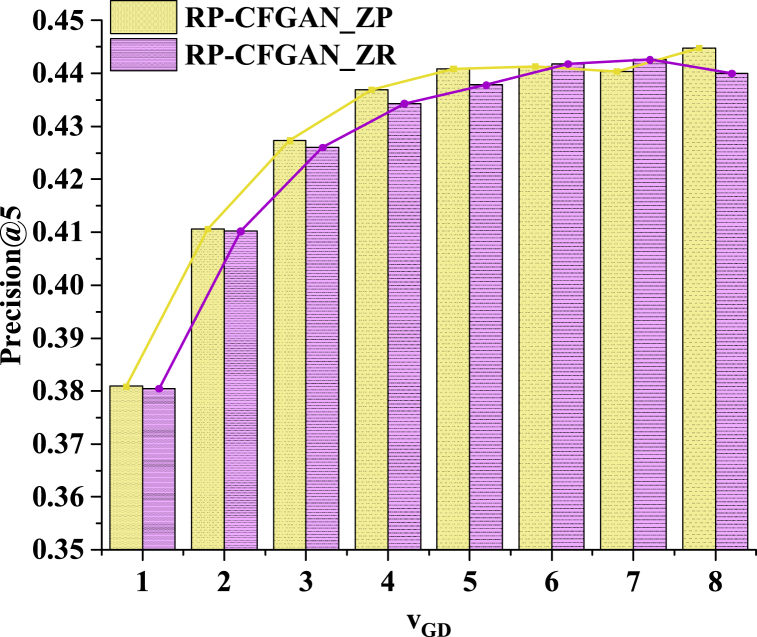
The influence of the training step ratio of G and D on the model.

Fixed $D_{step}$ is one. The value of $v_{GD}$ is changed by changing $G_{step}$. The results of P@5 are revealed in Fig. 5.

In Fig. 5, as the ratio $v_{GD}$ of training steps, the accuracy changes of both methods are very similar, both gradually increasing. When $v_{GD}$ = 6, the accuracy of the two methods tends to stabilize, and there is no remarkable improvement. Hence, the model's performance can be optimized by adjusting the ratio of training steps. At this point, the accuracy of RP-CFGAN_ZR and RP-CFGAN_ZP is 0.4418 and 0.4413, respectively. Therefore, at the same learning rate, G needs more training steps than D to compete with D. This is because generators typically require more training time and steps to learn complex data distributions and improve the quality of generated samples, while discriminators are relatively easier to enhance their ability to distinguish between real and generated samples with a small number of training steps. In summary, by adjusting the training step ratio of the generator and discriminator reasonably, the accuracy of the model can be effectively improved without changing the learning rate. This finding provides an important reference for the training optimization of DL models. Moreover, it validates the necessity of higher training step sizes required by the generator in processing complex data distributions in GANs. The two methods' results are similar, so subsequent studies only show the results of RP-CFGAN_ZP.

**Fig. 6:**
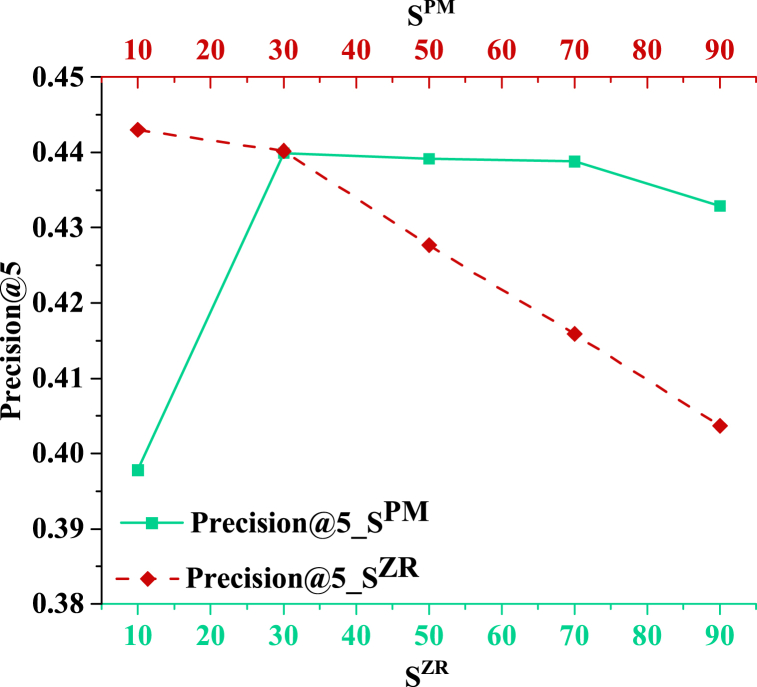
The impact of sampling ratio on the model.

$S^{PM}$ and $S^{ZR}$ are set to 30 to study the impact of $S^{ZR}$ and $S^{PM}$ on RP. Fig. 6 presents the results.

Fig. 6 demonstrates that when $S^{PM}$ is fixed, the model accuracy first increases and then decreases with the increase of $S^{ZR}$. When $S^{ZR}$ is 30, the model has the highest accuracy at 0.4399. A small $S^{ZR}$ indicates that the model uses a small number of negative samples, making it difficult to avoid the “all-1 strategy” situation. The model cannot appropriately avoid generating only all-1 outputs. In this case, the model cannot fully avoid generating all-1 outputs, thus being unable to accurately reflect users' diverse needs and personalized preferences. When the $S^{ZR}$ is too large, although the number of negative samples increases, the model may ignore users' personalized preferences. At this point, the model overly focuses on sample diversity and neglects personalized recommendations tailored to specific user needs, which can reduce the model's recommendation effectiveness. Notably, when $S^{PM}$ is small, the model can strike a balance between the two, considering the diversity of generated samples and the personalized needs of users. The results show that the sampling ratio has a marked effect on the model's performance. When the positive sample ratio is small, the model cannot avoid generating all-1 output. However, when the positive sample ratio is large, the model may ignore the user's personal preference. This is due to the smaller $S^{PM}$ allows the model to consider the samples' diversity and the user's personalized requirements, without being excessive towards a particular aspect. Consequently, it is necessary to choose the appropriate sampling ratio to balance the diversity of models and individual needs. In conclusion, selecting the appropriate sampling ratio for the model's performance is important. A moderate ratio of positive and negative samples can balance the diversity of models and individual needs, ensuring that the recommendation system can capture users' individual preferences while avoiding overly single output patterns.

**Fig. 7:**
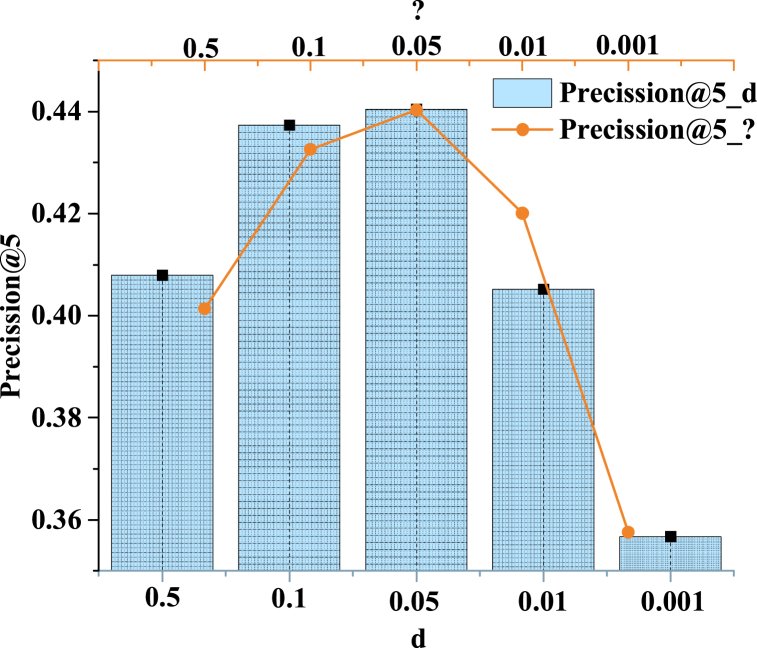
The impact of γ and δ on the model.

The impact of RP coefficients *γ* and *δ* on the model is shown in Fig. 7.

Fig. 7 shows that as δ decreases, the model's accuracy first increases and then decreases. When δ is 0.1 or 0.05, the model's accuracy is higher. When δ is too large, excessive emphasis is placed on generating values close to zero at the positions of all negative sampling samples. This is because δ is used as a penalty factor for “zero reconstruction”, which affects the model's tendency to generate negative samples. If δ is set too large, the generator is forced to generate near-zero output at the negative sample position to minimize the “zero reconstruction” penalty, which may result in the generated negative sample being too homogeneous and losing the original data distribution characteristics. In this case, the recommendation results of the model tend to be too consistent and fail to capture the diversified preferences of users, resulting in the lack of individuation and diversity of recommendation results. Conversely, overly diminutive values of δ attenuate the influence of negative samples excessively, rendering the generator ineffectual in circumventing the simplistic yet inefficient “all-1 strategy.” As a result, the generator may not be able to effectively learn and follow the complex distribution of real data, leading to a decline in the quality of the generated samples and difficulty in matching the user's real preferences and needs. Analogous to δ, either too large or too small γ can lead to a weakened model effect. Thus, in the experiment, a compromise value is chosen to set δ and γ to achieve the model's optimal effect. The results indicate that a proper RP coefficient can improve the model's accuracy, but too large or too small coefficients can lead to the degradation of the model's performance. Therefore, the RP coefficient should be selected carefully to optimize the model performance in practical applications.

#### 4.3.3 Comparison with traditional models

**Fig. 8:**
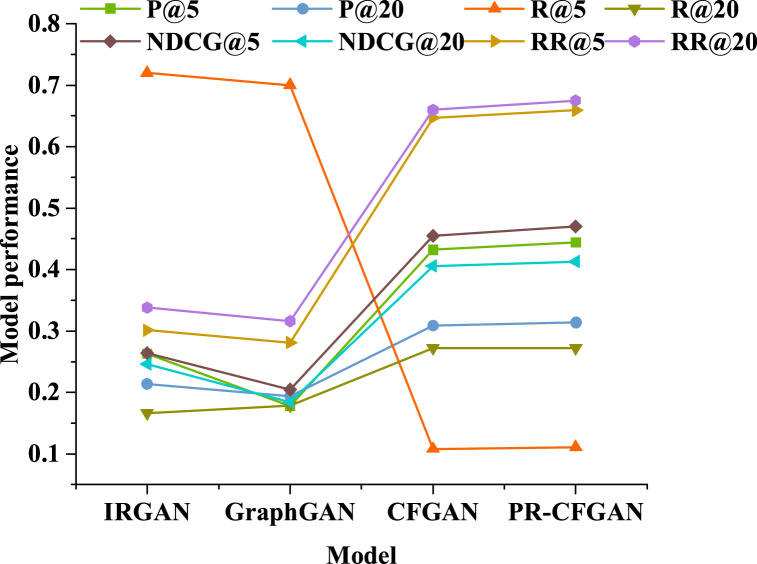
Comparison results of various models.

PR-CFGAN is compared with IRGAN, Graph GAN, and CFGAN at N values of 5 and 20, as demonstrated in Fig. 8.

Fig. 8 depicts that in Top20, compared to IRGAN, Graph GAN, and CFGAN, the accuracy of PR-CFGAN increases by 46.7 %, 61.9 %, and 1.62 %, while the other three indicators also show varying degrees of improvement. The results in the Top 5 are also the same, with a minimum improvement of 2.78 % in PR-CFGAN accuracy and a maximum improvement of 149.4 %. Hence, the PR-CFGAN model with embedded dual “1-Reconstruction” RP terms performs better. The findings reveal that compared with the traditional model, PR-CFGAN performs better in TopN recommendation, and the accuracy is remarkably improved. This illustrates that by embedding double “1-Reconstruction” RP items, the model's recommendation effect can be enhanced, thus promoting the model's practicality and applicability. This is because the double “1-Reconstruction” RP term effectively guides the generator to better simulate the real data distribution when generating samples by introducing a two-level penalty mechanism. The first “1-Reconstruction” penalty term guides the generator to accurately reconstruct the real data's features at the positive sample position, ensuring that the generated positive sample is of high quality and in line with the user's actual preference. The second “1-Reconstruction” penalty term guides the generator in some negative sample positions to avoid generating simple and non-diverse samples, to avoid the model falling into the problem of “all-1 strategy”, thus ensuring the diversity and authenticity of the generated samples. At the same time, the double “1-Reconstruction” RP term improves the model's sensitivity and understanding abilities to data distribution. Through the double penalty imposed on the generator in the training process, the model learns the complicated relationship between positive and negative samples in more detail. Hence, the user's personalized needs and preferences can be more accurately reflected when generating recommendation results, thus improving the accuracy and practicability of recommendations.

To further evaluate the proposed recommendation system (based on the RP-CFGAN model), a comparative analysis is made with several existing recommendation system models, encompassing the traditional CF model and the recently popular Machine Learning (ML)-Based recommendation system. This comparison focuses on a few key performance metrics: recommendation accuracy, and ability to and. The comparison results of recommendation system models are outlined in Table 2:

**Table 2:** Comparison results of recommender system models.

Model category	Recommendation accuracy	Data sparsity handle	Handling of cold start issues	User satisfaction
CF	3.6	2.9	2.8	3.7
ML	4.0	3.7	3.5	4.1
RP-CFGAN	4.3	4.5	4.4	4.5

Based on the results in Table 2, regarding recommendation accuracy, the RP-CFGAN model prominently outperforms the CF and ML models with a high score of 4.3. This indicates that the RP-CFGAN model is better able to accurately identify and predict user preferences, thereby recommending content that better meets their demands. Data sparsity poses a major challenge for recommendation systems, especially for new users or in cases with limited interactions. The RP-CFGAN model scores 4.5 on this metric, much higher than the CF and ML models, suggesting its ability to more effectively address data sparsity issues, possibly attributed to its GAN component, which improves the quality of recommendations by generating missing data. The cold start issue refers to how to provide effective recommendations in the absence of user historical data. The RP-CFGAN model also leads in this aspect with a score of 4.4, demonstrating its superiority over traditional models and ML-based models. The model may address the issue of recommending to new users by generating potential user preferences. User satisfaction, as an intuitive reflection of the success of a recommendation system, also receives the highest rating (4.5) in the RP-CFGAN model, reflecting high user approval and satisfaction with the recommended content. In summary, the recommendation system based on RP-CFGAN demonstrates significant advantages in addressing data sparsity, recommendation accuracy, and cold start issues, showing clear improvements compared to existing recommendation system models. Furthermore, based on the user satisfaction survey, users have a highly positive response to this system, proving its effectiveness and user acceptance in practical applications.

To assess the user acceptance of the recommendation system in practical applications, a user satisfaction survey is conducted. The group participating in this survey is college students, and the personal data involved follows strict privacy protection measures throughout the entire survey process to ensure the security and anonymity of audience information. In addition, the investigation process also follows ethical norms to ensure that the audience has sufficient informed consent to the investigation content and can withdraw from the investigation at any time. The survey is performed in the form of an online questionnaire, collecting a total of 800 valid responses. The main evaluation indicators include recommendation accuracy, interface friendliness, personalized experience, system response speed, and overall satisfaction. Satisfaction Is rated on a scale of 1–5, where 1 and 5 indicate very dissatisfied and very satisfied. The results of the user satisfaction survey are outlined in Table 3:

**Table 3:** The results of the user satisfaction survey.

Evaluation indicator	Average score	Standard deviation
Recommendation accuracy	4.2	0.8
Interface friendliness	4.4	0.7
Personalized experience	4.3	0.6
System response speed	4.1	0.9
Overall satisfaction	4.3	0.7

A long-term follow-up analysis is conducted for 6 months to evaluate the efficiency of the system. During this time, 500 college students are monitored for their use behavior and participation in physical activity. For each student, the frequency, duration, and participation in PE courses are recorded before and after the introduction of the recommendation system. At the same time, all data involved in the tracking and analysis process is processed with strict privacy protection to ensure that the collection and use of data comply with ethical requirements. The use of data is limited to the scientific purpose of this study and does not have any impact on the normal learning life of the audience. The results of the long-term follow-up analysis are shown in Table 4:

**Table 4:** The results of the long-term follow-up analysis.

Indicators	Average before recommendation	Recommended average	Change rate (%)
Participation frequency (times/week)	2.3	3.7	+60.9
Duration (minutes)	45	55	+22.2
Participation	3.5	4.4	+25.7
Course satisfaction (out of 5 points)	3.8	4.6	+21.1
Course completion rate	75 %	85 %	+13.3
Improvement of students' physical health index	60	68	+13.3
The proportion of self-selected courses	40 %	60 %	+50 %

In Table 3, Table 4, the results of the user satisfaction survey reveal that the satisfaction of users in various indicators exceeds 4 points, indicating that users have a high degree of recognition of the recommendation system. In particular, the score of interface friendliness and personalized experience is high, illustrating that the recommendation system can provide users with a comfortable and convenient experience. The results of long-term follow-up analysis show that PE course participation frequency, duration, and participation are markedly improved after the intervention of the recommendation system, and the participation frequency is the most significant increase, increasing by 60.9 %. This proves that the recommendation system not only increases students' interest in PE activities but also promotes their more frequent and active participation in PE courses. Based on the above analysis, it can be concluded that the PE course recommendation system based on the RP-CFGAN model presents high efficiency and good user acceptance in practical application, and effectively improves the PE activity participation level of students.

In addition, the increase in course satisfaction (+21.1 %) indicates that students are more satisfied with the content and quality of the recommended courses. Course completion rate increases from 75 % to 85 %, reflecting substantial improvements in course completion guided by the referral system. Particularly noteworthy is the improvement of students' physical health index (+13.3 %), which directly reflects the positive role of the recommendation system in promoting students' physical health. Moreover, the substantial increase in the proportion of self-selected courses (+50 %) proves that the system effectively promotes students' ability to choose courses according to their interests and needs, enhancing the initiative of learning and personalized experience. In summary, these detailed data confirm that the PE course recommendation system based on RP-CFGAN model has high efficiency and good user acceptance in practice. Furthermore, it verifies that students' participation levels in PE activities are effectively improved from multiple dimensions. At the same time, the system has a positive impact on promoting students' physical health, improving their learning satisfaction, and enhancing their course completion.

### 4.4 Discussion

In the context of the current integration of PE technologies, this study not only demonstrates the remarkable effectiveness of the PE course recommendation system based on the DL adversarial model (such as RP-CFGAN) in enhancing students' participation and interest. Moreover, this study further enriches the application of DL in the PE field. In addition, it significantly improves the accuracy and diversity of personalized recommendations by using the RP-CFGAN model, which is consistent with the application trend of AI in recommendation systems in recent years. Over the years, AI, especially DL technology, has made great progress in PE data analysis, sports skill assessment, and personalized training plan formulation [55]. In addition, researchers applied AI to recommendation systems and explored the basic methods and mainstream technologies in recommendation systems. They also studied how to effectively improve the technological development and application of recommendation systems through AI. Moreover, they reviewed the improvements made to recommendation systems through AI methods, such as GA, DL, fuzzy technology, transfer learning, active learning, and evolutionary algorithms, and neural networks [56]. On this basis, this study further improved the accuracy and diversity of personalized recommendations by introducing the RP-CFGAN model, fully reflecting the development trend of recommendation system technology for the past few years. The research examined the importance of recommendation systems in addressing information overload issues, particularly in areas like e-commerce, entertainment, and social media, guiding researchers and practitioners to understand new trends and challenges in this field [57]. Compared with this research, this study combined DL and IoT technology to solve the problem of personalized demand and sparse data in PE course recommendations, thus providing a more targeted and effective solution in the field of physical education. Some scholars comprehensively examined the latest research efforts on GNN-based recommendation systems and provided a classification method based on usage information types and recommendation tasks to classify GNN-based recommendation models. Besides, they systematically analyzed the challenges faced by applying GNN to different types of data and discussed how existing research in the field could address these challenges [58]. In contrast, this study not only applied the advanced RP-CFGAN model but also introduced the double “1-Reconstruction” RP term in the data processing process, markedly improving the accuracy and diversity of the recommendation system. In short, integrating DL algorithms into recommendation systems can greatly improve the accuracy of system recommendations. Compared with other studies, this study further discusses the latest progress in the field of recommendation systems. The recommendation system technology combined with AI can greatly enhance the system's recommendation accuracy, which is consistent with the previous research conclusions. Therefore, the results of this study are of great significance for improving the IoT's performance in PE course recommendation systems and provide a valuable reference for future research in the recommendation system field. However, there are still relatively few studies on its application in the PE course recommendation system, especially in combination with IoT technology to achieve dynamic and accurate recommendations.

The study findings depict that the DL-based PE course recommendation system in the context of IoT has significant potential to improve personalized recommendation accuracy and user satisfaction. Firstly, through IoT technology, the system can obtain students’ real-time physiological data, encompassing heart rate, body temperature, and exercise intensity, thereby better understanding their exercise habits, physical condition, and preferences. These data, combined with course data, enable the system to better understand students' physical condition and provide personalized course recommendations accordingly. For example, detecting an increased heart rate during a PE class may indicate that the student is interested or fully engaged in the course, allowing the system to recommend more related courses based on this information. This is consistent with the results of one study. In this study, IoT and DL are employed to improve the PE course recommendation system, and it can be observed that students' physiological data can help them obtain more personalized lessons [59]. However, compared with this, this study goes one step further and improves the performance of the recommender system in a sparse data environment through the realistic data generated by the GAN model, and solves the accuracy problem of the traditional recommender system when data is scarce.

Secondly, the use of GAN models further enhances the course recommendation system's performance. GAN models can generate realistic data, where the generator can produce outputs similar to real data, while the discriminator can accurately distinguish between real and generated data. One of the main reasons for choosing GANs in this study is to enhance the model's generative ability, with the generator generating course recommendations and the discriminator evaluating the quality of these recommendations, thus optimizing the recommendation system. Additionally, GANs can address sparsity and cold start issues in recommendation systems by filling gaps in user behavioral data and generating recommendations that better match user interests.

Compared with other similar studies, this study shows significant advantages in several aspects. For example, although the CF-based recommendation system proposed by scholars to some extent solved the problem of data sparsity, its effectiveness was limited when dealing with cold start problems [60]. Some studies improved personalized recommendations using recommendation systems based on deep neural networks, but the diversity of recommendations was still insufficient [61]. In contrast, this study introduces the RP-CFGAN model, which performs well in data sparsity and effectively overcomes the cold start problem, thereby remarkably improving the accuracy of recommendations. This indicates that the RP-CFGAN model has greater adaptability and flexibility in handling personalized recommendations. Experimental validation shows that the introduction of the dual “1-Reconstruction” RP-CFGAN model demonstrates advantages in recommendation accuracy. Compared to traditional CFGAN, RP-CFGAN can better handle users' personalized preferences, thereby improving the accuracy and diversity of recommendations. Moreover, the results of studying key hyperparameters suggest that effective performance improvement can be achieved by selecting appropriate hyperparameter settings. For instance, properly setting key parameters such as the training step ratio between the generator and discriminator, the penalty coefficients for “1-Reconstruction” and zero reconstruction, can significantly impact the model's performance. In short, the RP-CFGAN model and its hyperparameter optimization strategy represent a vital step toward the development of more intelligent and personalized recommendation systems, indicating a broad prospect for innovation in recommendation mechanisms in the educational technology domain.

Furthermore, in modern educational technology systems, especially when it comes to data processing with IoT devices, data security and privacy protection are critical. Integrating the RP-CFGAN model constructed in this study with IoT gadgets brings massive personal physiological data, such as heart rate, body temperature, and exercise intensity. The collection and processing of such data must strictly follow the standards of privacy protection and data security to prevent data leakage and improper use. First, to protect users' personal information, data encryption technology is implemented to encrypt sensitive data transmitted and stored to ensure that even if the data is intercepted, it cannot be read by unauthorized third parties. Secondly, to prevent the abuse and leakage of data, data anonymization and pseudo-anonymization technologies are adopted in the data processing process to separate personally identifiable information from behavioral data and avoid exposing the real identity of users in the analysis process. In addition, permission control mechanisms can be introduced to impose strict access control on different levels of users and system components, ensuring that only authorized personnel can access and process sensitive data. At the same time, in the process of model training, the principle of data minimization is implemented, that is, only the minimum amount of data needed to achieve the function of the system is collected and used to reduce privacy risks. To cope with possible security threats, regular system security assessment and vulnerability scanning can also be conducted to discover and patch potential security vulnerabilities promptly to protect the system from external attacks. In general, the implementation of the above privacy protection and security measures ensures the security and privacy of IoT devices and the RP-CFGAN model when processing user data, and provides users with secure and reliable personalized recommendation services.

In conclusion, this study is of great significance in theory and practice. From a theoretical point of view, by combining DL technology with the IoT, this study shows the application potential in the PE course recommendation system. IoT technology enables real-time access to students' physiological data, such as heart rate, body temperature, and exercise intensity, providing insight into their exercise habits, physical conditions, and preferences. This data, integrated with course data, enables the system to better understand students' physical conditions and personalize recommendations for appropriate courses based on real-time information. For example, the system can monitor a student's increased heart rate during PE class to determine whether the student is interested or engaged in the lesson, and then recommend more relevant lessons based on this information, which offers new possibilities and methods for personalized education. In theory, the RP-CFGAN model developed in this study can more accurately simulate users' actual needs when processing complex and dynamic datasets, thereby achieving more personalized recommendations. The innovation of this method lies in refining the model structure and optimizing the training process, enabling the system to maintain efficiency and accuracy even when facing large-scale and diverse data. Specifically, the introduction of the double “1-Reconstruction” RP term enables the generator to better generate samples that meet the actual needs of users. The discriminator can more effectively distinguish between real and generated data, thus improving the accuracy and practicality of the entire recommendation system. From a practical point of view, this study adopts the RP-CFGAN model as the core of the recommendation system, which markedly improves the accuracy and diversity of recommendations by introducing a double “1-Reconstruction” RP term. Compared with the traditional CFGAN model, RP-CFGAN can better handle the user's personalized preferences and optimize the recommendation results. Meanwhile, the experimental verification and optimization of key hyperparameters, such as the ratio of training steps between generator and discriminator, the penalty coefficient of “1-Reconstruction” and “zero reconstruction”, further improve the model's performance. These experimental results not only demonstrate the innovation and progress of recommendation system technology in the educational technology field but also provide a vital empirical basis and methodological guidance for the future development direction of recommendation systems. In the context of PE, the results of this study can be put into practice in many aspects. Firstly, PE workers can make use of the personalized course recommendation provided by the system to develop sports plans that are more in line with the actual needs of students, to improve the teaching effect and students' sports enthusiasm. Secondly, schools and educational institutions can integrate IoT technology and advanced recommendation algorithms to provide students with personalized PE course choices and enhance course engagement and satisfaction. Additionally, the research results can furnish data support for PE course designers and education decision-makers to help them understand the sports needs and preferences of different student groups, to optimize course and teaching strategies. In conclusion, this study not only promotes the application of recommendation system technology in the PE field but also offers a new method and tool for realizing personalized education. This theoretical innovation and practical exploration profoundly impact future related research and practical application, thus promoting the development and application of educational technology.

## 5 Conclusion

### 5.1 Research contribution

This study has achieved remarkable results by proposing a DL-based PE recommendation system under IoT and applying GANs to further optimize course recommendations. The experimental results reveal that the RP-CFGAN model with double “1-Reconstruction” PR terms has excellent performance in improving the accuracy of course recommendations. Specifically, the accuracy improvement of PR-CFGAN in the Top20 ranges from 46.7 % to 149.4 % compared to traditional models, which further demonstrates the effectiveness of dual “1-Reconstruction.” In addition, the experimental results also show that during training, G steps need to be more than D steps to achieve the best results. Moreover, the selection of sampling ratio and regularization coefficient also has an important impact on the model's performance and a trade-off between balancing model performance and personalized recommendation is needed.

The contribution lies in proposing and implementing a course recommendation system based on GAN. GAN serves as the core framework for the recommendation system, aiming to better simulate user behaviors and interests, thereby enhancing the accuracy and personalization of recommendations. Furthermore, this study optimizes the CFGAN by introducing a dual “1-Reconstruction” RP, creating a CF generative adversarial model. This optimization improves the accuracy of predicting student course preferences and adaptability, offering valuable insights for personalized recommendations and research within the field of recommendation systems.

## 6 Future works and research limitations

While the method proposed in this study has been elaborately described here, there are still some limitations. Firstly, although advanced technologies like DL and GAN are utilized, the method still heavily relies on the quality and availability of large-scale datasets. In practical applications, insufficient and unrepresentative data may affect the model's performance and recommendation accuracy. Secondly, the recommendation results of this method may be influenced by data collection devices and environments, such as the accuracy and reliability of smart wearable devices, as well as data noise and interference in IoT environments. These factors may lead to biased or inaccurate recommendation results. Additionally, the consideration of user privacy and data security in this method seems insufficient. Although the importance of privacy protection and data security is mentioned, specific privacy protection mechanisms and security measures are not thoroughly discussed, which may raise concerns and opposition from users in practical applications. Therefore, future research should focus on the following directions. First, the structure of the RP-CFGAN model can be further optimized to improve the recommendation system's efficiency and accuracy. Second, the research scope should be expanded to explore the impact of more types of IoT device data on sports course recommendation, thus improving the recommendation system's personalized level. At the same time, it can consider integrating other DL models or traditional recommendation algorithms to enhance the effect of course recommendation. Future research should also pay more attention to data privacy protection and information security to ensure the sustainable development of the recommendation system. In addition, it should strengthen the cooperation with educational institutions and PE training organizations, make full use of their professional knowledge and resources, and jointly promote the PE course recommendation system's development and application. The exploration in the above directions can lay the foundation for realizing a more intelligent and personalized PE course recommendation system.

The significance and implications of the research results are multifaceted. First, by combining IoT technology with DL, a more intelligent and personalized service is provided to the PE course recommendation system. This not only improves students' learning and sports experience but also provides new ideas and new methods for the application of technology in the education field and promotes the development of education informatization. This finding shows that the application of modern technology in education not only meets the individual needs of students but also optimizes the allocation of teaching resources and course design based on a data-driven way, which offers strong support for the fine management of education. Second, this study effectively solves the common problems of data sparsity and cold start in traditional recommendation methods by using advanced models such as GAN, thereby enhancing the accuracy and personalization level of the recommendation system. This not only has important reference value for the design and optimization of online education platforms but also provides theoretical support and practical guidance for improving recommendation systems in other fields such as e-commerce, social media, etc. This indicates that DL and GAN have unique advantages in handling complex and dynamic user data, enabling more accurate prediction of user needs and providing more targeted recommendations, thereby improving user satisfaction and engagement. Moreover, through comparative analysis of the experimental results, it can be found that the improved recommendation model exhibits significant advantages in performance, which offers useful reference and guidance for future related research. Specifically, these research results not only validate the model's effectiveness in specific application scenarios but also provide inspiration for other researchers to explore combinations of different methods and models. For example, researchers can further explore how to use similar methods in other types of datasets or application areas to improve the recommendation system's performance. The study also identifies some limitations and potential issues that are not mentioned, which should be given sufficient attention in subsequent research. For example, issues such as data privacy protection, system scalability, and adaptability in various application scenarios are vital topics that need to be further explored in future research. Subsequent research can evaluate and improve this method from multiple perspectives to enhance the performance and user experience of the recommendation system. These improvements not only help to improve the existing recommendation system but also provide a theoretical basis and practical support for the wide application of technology in many fields such as education, health management, and personalized service.

## Data availability statement

Data will be made available on request.

## CRediT authorship contribution statement

**Aiyuan Zhen:** Writing – original draft, Resources, Project administration, Methodology, Formal analysis, Data curation, Conceptualization. **Xin Wang:** Writing – review & editing, Visualization, Validation, Supervision, Software, Resources.

## Declaration of competing interest

The authors declare that they have no known competing financial interests or personal relationships that could have appeared to influence the work reported in this paper.
